# Diagnostic performance of artificial intelligence–enabled electrocardiography for pulmonary hypertension: a systematic review and meta-analysis

**DOI:** 10.1093/ehjdh/ztag143

**Published:** 2026-09-08

**Authors:** Karan Raj Nazareth, Eyad Jamileh, Fahad Muhammad, Omar Farooq, Anisha Sequeira, Alishba Awais, Ibrahim Antoun

**Affiliations:** School of Medicine, University of Buckingham, Buckingham, UK; School of Medicine, Queens University Belfast, Belfast, UK; Department of Medicine, Furness General Hospital, University Hospitals Morecambe Bay NHS Foundation Trust, Cumbria, UK; School of Medicine, Queen Mary University of London, London, UK; Department of Medicine, Royal Blackburn Teaching Hospital, East Lancashire Hospitals NHS Trust, Blackburn, UK; School of Medicine, University of Buckingham, Buckingham, UK; School of Medicine, University of Buckingham, Buckingham, UK; Department of Cardiology, University Hospitals of Leicester NHS Trust, Glenfield Hospital, Groby Road, Leicester LE3 9QP, UK; Divison of Cardiovascular Sciences, Clinical Science Wing, University of Leicester, Glenfield Hospital, Groby Road, Leicester LE3 9QP, UK

**Keywords:** Artificial intelligence, Electrocardiography, Pulmonary hypertension, Deep learning, Diagnostic accuracy

## Abstract

Pulmonary hypertension (PH) is a progressive, under-recognized condition with substantial morbidity and mortality. Its non-specific presentation and multimodal diagnostic pathway contribute to delayed diagnosis. Artificial intelligence (AI)-enabled electrocardiography (ECG) may offer a scalable approach to case finding. This PRISMA-DTA systematic review and meta-analysis was registered in PROSPERO (CRD420251241142). MEDLINE, Embase, Web of Science, Scopus, and IEEE Xplore were searched through July 2026, with Google Scholar and citation searching used as supplementary sources. Eligible studies evaluated AI-enabled ECG in adults against right-heart catheterization (RHC)-confirmed PH or echocardiographic PH-related phenotypes. Two reviewers independently screened studies, extracted data, and assessed risk of bias using QUADAS-2. One ECG-only area under the receiver operating characteristic curve (AUROC) per study was pooled using a random-effects model. Seven retrospective studies were included; six contributed to meta-analysis. The pooled AUROC was 0.885 (95% CI 0.855–0.915), with very high heterogeneity (I² = 98.68%; *P* < 0.0001). The estimate combined heterogeneous RHC, echocardiographic, and clinical-echocardiographic definitions and therefore did not represent accuracy for uniformly RHC-confirmed PH. Four studies performed external validation. Multimodal models outperformed ECG alone in two studies but were excluded from pooling. AI-enabled ECG shows promising discrimination for PH-related targets, but current evidence is retrospective and heterogeneous. It should be considered an investigational case-finding or triage aid rather than a definitive diagnostic stand-alone rule-out test.

## Graphical Abstract

**Graphical Abstract:**
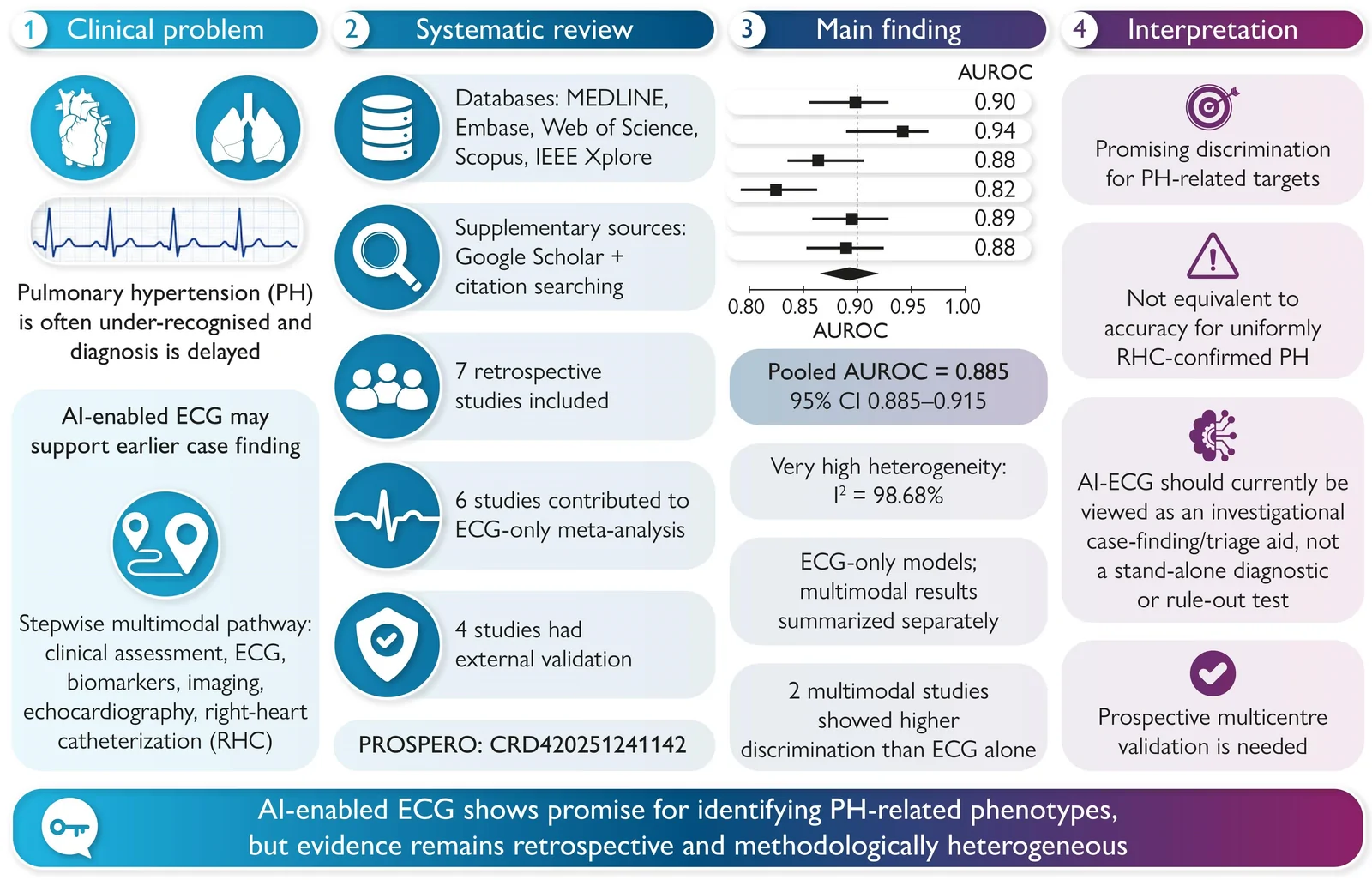

## Introduction

Pulmonary hypertension (PH) is a complex, progressive pathophysiological disorder defined haemodynamically by a resting mean pulmonary arterial pressure of >20 mmHg measured during right-heart catheterization (RHC).^1^ It affects ∼1% of the global population, with prevalence increasing among older adults and people with underlying cardiac or pulmonary disease.^2^ Pulmonary hypertension commonly presents with non-specific symptoms, including dyspnoea, fatigue, and oedema, and diagnostic delays exceeding 2 years have been reported.^3,4^ Delayed recognition is clinically important because patients may present with advanced pulmonary vascular disease, right ventricular failure, and a poorer prognosis.

Current guidelines recommend a stepwise, multimodal diagnostic pathway incorporating clinical assessment; electrocardiography (ECG); biomarkers, such as brain natriuretic peptide (BNP) or N-terminal pro-BNP; pulmonary function assessment; and thoracic imaging. Echocardiography estimates the probability of PH and evaluates cardiac structure and function, whereas RHC is required for definitive haemodynamic confirmation and classification.^1^ The 12-lead ECG is inexpensive, widely available, and routinely performed, but conventional signs of right-heart pressure overload, including right-axis deviation, P pulmonale, and right-ventricular hypertrophy, have limited sensitivity and often emerge only in more advanced disease.

Deep-learning methods can extract subtle, high-dimensional patterns from raw ECG waveforms that are not apparent during conventional interpretation. Artificial intelligence (AI)–enabled ECG has already shown utility for identifying conditions not traditionally diagnosed by ECG, including occult left-ventricular systolic dysfunction.^5^ These advances have prompted the development of ECG-based models intended to identify PH or related echocardiographic phenotypes before definitive investigation.

We therefore conducted a diagnostic test accuracy systematic review and meta-analysis to evaluate AI–enabled ECG models for PH-related target conditions. The objectives were to summarize study populations, reference standards, AI architectures, and validation strategies; quantify ECG-only discrimination where appropriate; and examine the methodological and clinical heterogeneity that affects interpretation and potential implementation.

## Methods

### Protocol and reporting

The review was conducted in accordance with the Preferred Reporting Items for Systematic Reviews and Meta-Analyses of Diagnostic Test Accuracy Studies.^6^ The protocol was prospectively registered with PROSPERO on 5 February 2025 (CRD420251241142).^7^

### Search strategy and information sources

MEDLINE via PubMed, Embase via Ovid, Web of Science Core Collection, Scopus, and IEEE Xplore were searched from database inception to July 2026. The strategy combined controlled vocabulary and free-text terms across three concepts: (i) PH, pulmonary arterial hypertension, or elevated pulmonary artery pressure; (ii) ECG, electrocardiogram, or EKG; and (iii) AI, machine learning, deep learning, neural networks, convolutional neural networks, or algorithms. Synonyms were combined using OR, and the three concept groups were intersected using AND. Syntax was adapted for each database. No language or publication-date restrictions were applied.

Google Scholar was used as a supplementary source for cross-disciplinary reports and citations not retrieved by the formal database searches. The exact query was run on 27 July 2026, and the first 200 results ranked by relevance were screened. This pragmatic limit was used because Google Scholar does not support stable, exhaustive retrieval or reproducible bulk export. Reference lists and forward citations of included studies and relevant reviews were also screened. Complete database-specific strategies, search dates, and numbers retrieved are provided in Supplementary material online, *Table S1*.

### Eligibility criteria

Studies were eligible if they (i) included adults aged at least 18 years; (ii) applied an AI, machine-learning, or deep-learning model to ECG data to detect PH or a PH-related phenotype; (iii) used either RHC-confirmed PH or a study-defined echocardiographic phenotype representing elevated pulmonary pressure or echocardiographic probability of PH as the target condition; and (iv) reported an area under the receiver operating characteristic curve (AUROC), sensitivity, specificity, or other diagnostic performance measure.

Echocardiography-labelled studies were included because the proposed role of AI–enabled ECG is preliminary case finding or triage in populations for whom routine RHC would not be appropriate. In such studies, the target was elevated estimated pulmonary pressure or echocardiographic probability warranting further evaluation, rather than definitive PH. Echocardiographic surrogate labels were therefore distinguished from RHC-confirmed PH throughout data extraction, quality assessment, synthesis, and interpretation. Each study was classified as RHC based, echocardiography based, mixed RHC/echocardiography, or clinical–echocardiographic according to the criteria used to generate outcome labels.

### Study selection and data extraction

Two reviewers independently screened titles and abstracts, assessed potentially eligible full texts, and extracted data using a standardized form. Disagreements were resolved by discussion and consensus.

Extracted variables included study design and setting; population; numbers of unique patients and ECG examinations; prevalence and definition of the target condition; RHC and echocardiographic criteria; ECG-to-reference-standard interval; ECG lead configuration, duration, and sampling frequency; model input and preprocessing; AI architecture; training, validation, and test strategy; repeated-ECG handling; patient-level separation; external validation; threshold selection; and reported diagnostic performance estimates. We specifically recorded whether all ECGs belonging to one participant remained within a single data partition, because participant overlap between training and validation data may lead to information leakage and overoptimistic performance.

### Quality and applicability assessment

Two reviewers independently assessed the risk of bias and applicability using QUADAS-2.^8^ The standard domains were patient selection, index test, reference standard, and flow and timing. The assessment was tailored to AI diagnostic studies.

Particular attention was given to the reference-standard domain. We extracted the exact haemodynamic or echocardiographic criteria, whether thresholds were prespecified, the interval between ECG and reference assessment, and whether the reference standard was interpreted independently of the AI output. Echocardiography-based labels were considered potential sources of disease misclassification and applicability concerns because they identify elevated estimated pressure or echocardiographic probability rather than definitive PH. In mixed-reference studies, we assessed whether RHC was prioritized when both RHC and echocardiography were available.

Potential data leakage was considered in the index-test and flow-and-timing domains. Patient-level separation was judged adequate when all examinations from an individual were explicitly retained within one partition or when only one ECG per patient was used. Where repeated ECGs were included but separation between cross-validation folds was not reported, the risk was considered unclear.

### Statistical analysis

Quantitative synthesis was undertaken where compatible data were available. Area under the receiver operating characteristic curves were pooled using a random-effects inverse-variance model in MedCalc (MedCalc Software Ltd, Ostend, Belgium). Standard errors were derived from reported 95% CIs. Between-study heterogeneity was evaluated using Cochran’s Q and quantified using I^2^. Small-study effects were not formally assessed because fewer than 10 studies contributed to the synthesis, making funnel-plot interpretation and statistical tests of asymmetry unreliable.

A bivariate random-effects meta-analysis of sensitivity and specificity was not undertaken because 2 × 2 data were reported inconsistently, operating thresholds varied, and reference standards and target definitions were heterogeneous. Sensitivity, specificity, and predictive values were therefore summarized descriptively.

Only ECG-only model outputs were eligible for the primary meta-analysis. Multimodal estimates incorporating chest radiography, BNP, or other modalities were summarized separately. To avoid correlated double counting, one ECG-only AUROC was selected per study using a *post hoc* hierarchy: (i) the study’s primary overall outcome rather than a PH subtype or alternative model output; (ii) independent external validation over internal validation when a compatible precision estimate was available; (iii) an independent test estimate over development performance; and (iv) contemporaneous diagnostic assessment over earlier prediagnostic time points. Where an external AUROC lacked a CI or other measure from which a standard error could be derived, the most robust patient-level internal estimate with extractable precision was selected.

Reference standards were classified at the study level. A formal RHC-only subgroup meta-analysis was not performed because only one study reported a separately extractable RHC-specific AUROC with corresponding precision. The available within-study comparison by reference-standard type was reported narratively.

## Results

### Study selection and overall characteristics

A total of 1640 records were screened, 22 full-text articles were assessed, and seven retrospective observational studies were included (*Figure 1*). These were C.M. Liu *et al*.,^9^ Kishikawa *et al*.,^10^ Aras *et al*.,^11^ DuBrock *et al.*,.^12^ P.Y. Liu *et al*.,^13^ Tison *et al*.,^14^ and Kwon *et al*.^15^

**Figure 1:**
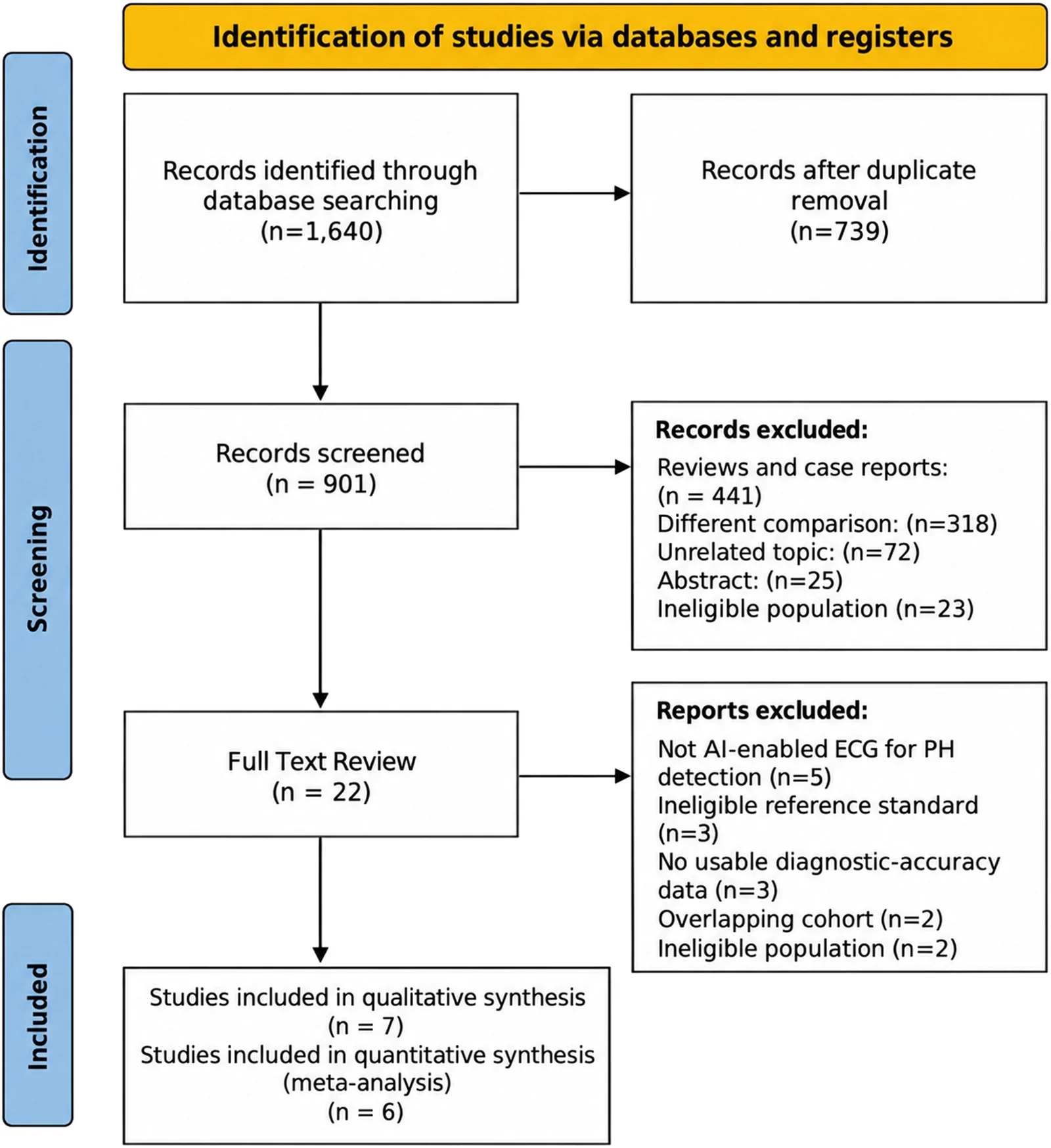
PRISMA flow chart.

Study size and structure varied substantially. Some investigations used one ECG per patient, whereas others included repeated examinations, producing substantially more ECGs than unique participants. All seven studies reported an ECG-only output. Five primarily evaluated ECG-based models, while two additionally assessed multimodal approaches: Kishikawa *et al*.^10^ combined ECG, chest radiography, and BNP, and P.Y. Liu *et al*.^13^ combined ECG and chest radiography. Only ECG-only estimates were eligible for the primary meta-analysis.

### Target conditions and reference standards

Target-condition definitions differed considerably (*Table 1*). C.M. Liu *et al*.^9^ defined elevated pulmonary artery pressure as TTE-estimated pulmonary artery pressure > 50 mmHg. Kishikawa *et al*. used right-ventricular systolic pressure ≥ 40 mmHg.^9^ Aras *et al*. and DuBrock *et al*.^11,12^ prioritized RHC where available, using mean pulmonary arterial pressure > 20 mmHg, but otherwise applied high-probability echocardiographic tricuspid regurgitation velocity thresholds. P.Y. Liu *et al*.^13^ used TTE-estimated systolic pulmonary artery pressure > 50 mmHg. Tison *et al*.^14^ used a clinical–echocardiographic pulmonary arterial hypertension phenotype requiring an echocardiographic indication of disease and PAH-specific treatment. Kwon *et al*.^15^ used a composite echocardiographic definition incorporating estimated pulmonary pressure and additional right-ventricular findings.

**Table 1:** Study-level definitions of pulmonary hypertension and handling of the reference standard

Study	Positive target definition	Negative/control definition	ECG-reference-standard interval	Reference-standard classification
C.M. Liu *et al*.^8^	Elevated pulmonary artery pressure, defined as TTE-estimated PAP > 50 mmHg, calculated as 4 × TRV^2^ plus estimated right-atrial pressure	TTE-estimated PAP ≤ 50 mmHg	First eligible ECG–TTE pair within 2 weeks	Echocardiography-only surrogate target
Kishikawa *et al*.^9^	RVSP ≥ 40 mmHg on TTE, calculated as 4 × TRV^2^ plus estimated right-atrial pressure	RVSP < 40 mmHg	Echocardiographic assessment within 1 year of ECG	Echocardiography-only surrogate target
Aras *et al*.^10^	RHC mPAP > 20 mmHg; when RHC was unavailable, peak TRV > 3.4 m/s on TTE	RHC mPAP ≤ 20 mmHg; when RHC was unavailable, TRV ≤ 2.8 m/s	Nearest ECG within 90 days of RHC or TTE	Mixed RHC/echocardiography, with RHC prioritized
DuBrock *et al*.^11^	At least one resting RHC mPAP > 20 mmHg; in patients without RHC, TRV > 3.4 m/s on TTE	All available RHC mPAP values ≤ 20 mmHg; in patients without RHC, all TRV values ≤ 2.8 m/s	Diagnostic ECG within 30 days of the index assessment	Mixed RHC/echocardiography, with RHC prioritized
P.Y. Liu *et al*.^12^	TTE-estimated systolic PAP > 50 mmHg, corresponding to peak TRV > 3.4 m/s	TTE-estimated systolic PAP ≤ 50 mmHg	ECG within 7 days of TTE	Echocardiography-only surrogate target
Tison *et al*.^13^	Echocardiographic indication of PAH together with treatment using PAH-specific medication; echocardiograms were secondarily reviewed	Age-, sex-, race-, and study-year-matched controls without the study-defined PAH phenotype	No uniform ECG–echocardiography interval reported for the PAH analysis	Clinical–echocardiographic PAH phenotype; not uniformly RHC-confirmed
Kwon *et al*.^14^	TR peak gradient > 32 mmHg, estimated PA systolic pressure > 35 mmHg, or TR peak gradient ≤ 32 mmHg with at least two of the following: TAPSE < 16 mm, RV fractional area change < 35%, tricuspid annular systolic velocity < 10 cm/s, or RV dilatation	Absence of the study-defined echocardiographic PH criteria	ECG and echocardiography within 4 weeks	Echocardiography-only composite target

RHC provides definitive haemodynamic confirmation of PH. Echocardiography-based outcomes represent study-specific surrogate target conditions, including elevated estimated pulmonary pressure or echocardiographic probability of PH, and should not be interpreted as definitive PH diagnoses. Intermediate TRV values between 2.8 and 3.4 m/s were excluded by Aras *et al*.; DuBrock *et al*. similarly separated high- and low-probability echocardiographic groups.

mPAP, mean pulmonary arterial pressure; PA, pulmonary artery; PAH, pulmonary arterial hypertension; PAP, pulmonary artery pressure; PH, pulmonary hypertension; RHC, right-heart catheterization; RV, right ventricle; RVSP, right-ventricular systolic pressure; TAPSE, tricuspid annular plane systolic excursion; TR, tricuspid regurgitation; TRV, tricuspid regurgitation velocity; TTE, transthoracic echocardiography.

The positive target classes therefore represented related but non-equivalent phenotypes, including haemodynamically confirmed PH, elevated estimated pulmonary pressure, echocardiographic probability of PH, and a selected PAH phenotype.

### AI models, validation and repeated electrocardiography

AI methods also varied substantially (*Table 2*). Aras *et al*., DuBrock *et al*., Kishikawa *et al*., and P.Y. Liu *et al*. used end-to-end convolutional neural networks applied to raw waveform data.^10–13^ Kwon *et al*.^15^ combined a raw-waveform convolutional neural network with a deep neural network using demographic and conventional ECG variables. Tison *et al*.^14^ used convolutional neural-network and hidden-Markov-model segmentation to derive a 725-element interpretable ECG profile, followed by a gradient-boosting classifier. C.M. Liu *et al*.^9^ used a deep neural-network ECG model adapted from a previously developed arrhythmia architecture. Independent external validation was performed in four studies.^9,12,13,15^

**Table 2:** AI model characteristics, validation strategies, and handling of repeated ECG examinations

Study	Participants and ECG examinations	ECG input and AI model	Data partitioning and repeated-ECG handling	Independent external validation
C.M. Liu *et al*.^8^	41 097 patients; first eligible ECG–TTE pair per patient. External Japanese cohort: 279 patients	Raw 12-lead ECG; deep neural network architecture adapted from a previously developed ECG–arrhythmia model	Ten-fold 8:1:1 training–validation–test procedure. Each patient appeared in only one partition and was tested once across the rolling folds	Yes; two Japanese hospitals
Kishikawa *et al*.^9^	71 826 patients and 123 260 ECG examinations	Raw 12-lead ECG; 10-s recordings sampled at 500 Hz; seven-convolutional-layer CNN	Training, validation, and test allocation of 7:1.5:1.5 using unique patient identifiers. All examinations from one patient remained in the same partition	No independent external cohort; multicentre data from seven Japanese institutions were randomly partitioned
Aras *et al*.^10^	24 470 patients: 5016 PH and 19 454 non-PH; one ECG selected per patient	Raw 10-s 12-lead ECG sampled at 250 or 500 Hz and converted to a 2500 × 12 matrix; 15-layer one-dimensional residual CNN	Random patient-level 7:1:2 training–development–test split; one index ECG per patient	No
DuBrock *et al*.^11^	Mayo cohort: 39 823 PH-likely and 219 404 controls; external Vanderbilt cohort: 6045 PH-likely and 24 256 controls	Raw 10-s 12-lead ECG resampled to 500 Hz; single-branch one-dimensional CNN trained using waveform crops	Mayo patients were allocated to mutually exclusive training, validation, and test sets in proportions of 48%, 12%, and 40%. One ECG per patient was used within each diagnostic or prediagnostic test window	Yes; Vanderbilt University Medical Center
P.Y. Liu *et al*.^12^	Hospital A, 85 193 patients; Hospital B, 16 736 patients. Development ECG data included 72 948 ECGs from 36 851 patients	Raw 10-s 12-lead ECG sampled at 500 Hz; CNN with 82 trainable layers. ECG and CXR model outputs were combined using extreme gradient boosting	Patients were randomly assigned to development, tuning, or internal validation sets. Each patient’s data were exclusive to one partition	Yes; independent community hospital
Tison *et al*.^13^	525 PAH case ECGs and 1764 matched control ECGs; counts represented ECGs rather than clearly reported unique participants	CNN and hidden Markov model used for ECG segmentation; 725-element interpretable ECG profile; gradient-boosting classifier	Held-out predictions from five-fold cross-validation. Multiple ECGs from case patients were eligible, but whether all ECGs from one patient remained within one fold was not reported	No
Kwon *et al*.^14^	Derivation: 56 670 ECGs from 24 202 patients; internal validation: 3174 patients/ECGs; external validation: 10 865 patients/ECGs	Ensemble comprising a five-hidden-layer DNN using demographic and conventional ECG variables and a two-dimensional CNN using a 12 × 4000 raw-waveform matrix	Repeated ECGs were used during derivation. Internal and external validation used one ECG per patient. Derivation and internal validation populations were separated according to follow-up echocardiography status	Yes; independent hospital

AI, artificial intelligence; CNN, convolutional neural network; CXR, chest radiography; DNN, deep neural network; ECG, electrocardiogram; PAH, pulmonary arterial hypertension; PH, pulmonary hypertension; TTE, transthoracic echocardiography.

Handling of repeated ECGs differed. Aras *et al*.^11^ used one ECG per patient. C.M. Liu *et al*. selected the first eligible ECG–TTE pair and ensured that each patient appeared in only one cross-validation partition.^8^ Kishikawa *et al*. and P.Y. Liu *et al*.^10,13^ assigned all examinations from an individual to one partition using patient identifiers. DuBrock *et al*.^12^ used mutually exclusive patient-level development and test sets and one ECG per patient for each test window. Kwon *et al*.^15^ used repeated ECGs during derivation but one ECG per patient in internal and external validation. Tison *et al*.^14^ used all available ECGs from PAH cases and five-fold cross-validation, but did not report whether multiple ECGs from the same patient remained within one fold; data leakage could therefore not be excluded.

### Quality assessment

Methodological quality varied. Patient-selection bias was frequently unclear or high because all studies were retrospective and several used selected or non-consecutive cohorts. Index-test risk was generally low to unclear, with incomplete reporting of threshold selection, calibration, and blinding in some studies.

The reference-standard domain was a principal source of concern. Four studies used exclusively echocardiographic targets,^9,10,13,15^ one used a clinical–echocardiographic phenotype,^14^ and two used mixed RHC/echocardiographic definitions.^11,12^ Echocardiography-only studies were considered to have high applicability concerns for questions concerning confirmed PH because they evaluated surrogate pressure or probability labels. Flow-and-timing concerns arose where the permitted ECG-to-reference interval was long or incompletely reported and where repeated-ECG handling was unclear.

### Diagnostic performance and selection of area under the receiver operating characteristic curves

Six studies contributed one ECG-only AUROC to the quantitative synthesis; the selected estimates and reasons for their selection are presented in *Table 3*. Kwon *et al*. and DuBrock *et al*.^12,15^ contributed external-validation diagnostic estimates. Aras *et al*. and Kishikawa *et al*. contributed their primary independent test estimates.^11,12^ Tison *et al*.^14^ contributed the held-out five-fold cross-validation estimate. C.M. Liu *et al*. contributed the patient-level ten-fold cross-validation estimate because its external cohort reported the same AUROC of 0.88 without a CI. P.Y. Liu *et al*.^9^ was excluded from quantitative pooling because the study did not report a CI, standard error, or another compatible measure of precision for its ECG-only AUROC.^13^

**Table 3:** Study-level ECG diagnostic performance and selection of AUROC estimates for meta-analysis

Study	Principal ECG-only validation result(s)	Sensitivity	Specificity	AUROC selected for meta-analysis	Rationale for selection
C.M. Liu *et al*.^8^	Ten-fold cross-validation: AUROC 0.880 (95% CI 0.870–0.890). External validation: AUROC 0.88; CI not reported	81.0% internally; 93.9% externally	79.6% internally; 58.7% externally	0.880 (95% CI 0.870–0.890)	Patient-level cross-validation estimate with extractable precision; external AUROC lacked a compatible CI or SE
Kishikawa *et al*.^9^	ECG-only test AUROC 0.818 (95% CI 0.808–0.828)	75.6%	72.5%	0.818 (95% CI 0.808–0.828)	Primary independent ECG-only test estimate
Aras *et al*.^10^	Overall PH test AUROC 0.890 (95% CI 0.880–0.900). RHC-labelled AUROC 0.89; echocardiography-labelled AUROC 0.90	79% overall; ∼78–79% by reference-standard subgroup	84%	0.890 (95% CI 0.880–0.900)	Primary overall PH outcome from the independent test set
DuBrock *et al*.^11^	Diagnostic AUROC 0.920 at Mayo and 0.880 externally at Vanderbilt	85.5% at Mayo; 82.3% at Vanderbilt	83.8% at Mayo; 77.0% at Vanderbilt	0.880 (95% CI 0.870–0.880)	Independent external contemporaneous diagnostic cohort
P.Y. Liu *et al*.^12^	ECG-only AUROC 0.8261 internally and 0.8348 externally	76.6% internally; 78.9% externally	74.5% internally; 73.7% externally	Not included	No reported CI, SE, or other compatible measure of AUROC precision
Tison *et al*.^13^	Held-out five-fold cross-validation AUROC 0.940 (95% CI 0.930–0.950)	Not reported for the PAH model	Not reported for the PAH model	0.940 (95% CI 0.930–0.950)	Principal held-out cross-validation estimate
Kwon *et al*.^14^	Internal AUROC 0.859 (95% CI 0.855–0.863); external AUROC 0.902 (95% CI 0.900–0.905)	80% internally and externally	74% internally; 84% externally	0.902 (95% CI 0.900–0.905)	Independent external-validation estimate

One ECG-only AUROC was selected per study to avoid correlated double counting. Selection prioritized the primary overall target, independent external validation where an estimate of precision was available, independent test performance over development performance, and contemporaneous diagnostic assessment over earlier prediagnostic time points. Multimodal outputs were excluded from the ECG-only meta-analysis. Kishikawa *et al*. additionally reported an AUROC of 0.872 (95% CI 0.829–0.915) for its ECG–CXR–BNP ensemble, while P.Y. Liu *et al*. reported an external AUROC of 0.8734 for its combined ECG–CXR model.

AUROC, area under the receiver operating characteristic curve; BNP, brain natriuretic peptide; CI, confidence interval; CXR, chest radiography; ECG, electrocardiogram; PAH, pulmonary arterial hypertension; PH, pulmonary hypertension; RHC, right-heart catheterization; SE, standard error.

Selected ECG-only AUROCs ranged from 0.818 to 0.940. Tison *et al*.^14^ reported an AUROC of 0.940 (95% CI 0.930–0.950). Kwon *et al*.^15^ reported 0.859 (95% CI 0.855–0.863) internally and 0.902 (95% CI 0.900–0.905) externally. The external diagnostic AUROC in DuBrock *et al*.^12^ was 0.880, compared with 0.920 in the Mayo Clinic test cohort. C.M. Liu *et al*.^9^ reported an AUROC of 0.880 (95% CI 0.870–0.890), and Aras *et al*.^11^ reported 0.890 for the overall PH target.

In Kishikawa *et al*., the ECG-only convolutional neural network achieved an AUROC of 0.818 (95% CI 0.808–0.828), which was included in the meta-analysis. The ECG-chest-radiography-BNP ensemble achieved an AUROC of 0.872 (95% CI 0.829–0.915) and was excluded from the ECG-only synthesis.^10^ P.Y. Liu *et al*.^13^ reported ECG-only AUROCs of 0.8261 internally and 0.8348 externally; the corresponding combined ECG-chest-radiography AUROCs were 0.8644 and 0.8734.

### Reference-standard sensitivity analysis

No primary study-level estimate represented a uniformly RHC-confirmed PH population. Aras *et al*.^11^ reported similar discrimination in the RHC-labelled cohort (AUROC 0.89, 95% CI 0.87–0.90) and the echocardiography-labelled cohort (AUROC 0.90, 95% CI 0.89–0.91). DuBrock *et al*.^12^ used RHC where available, but 66% of the Mayo PH-likely cohort was classified using an echocardiographic tricuspid regurgitation velocity threshold; restriction to RHC-defined PH did not materially reduce performance. Because only one independently extractable RHC-specific AUROC with corresponding precision was available, an RHC-only meta-analysis was not statistically or clinically appropriate.

### Quantitative synthesis of area under the receiver operating characteristic curve

Across six studies contributing one ECG-only estimate each, the random-effects pooled AUROC was 0.885 (95% CI 0.855–0.915) (*Figure 2*). Between-study heterogeneity was very high (I^2^ = 98.68%; Q = 379.86; *P* < 0.0001). The synthesis combined mixed RHC/echocardiographic, echocardiography-only, and clinical–echocardiographic target definitions and should therefore not be interpreted as the diagnostic accuracy of AI-enabled ECG for uniformly RHC-confirmed PH.

**Figure 2:**
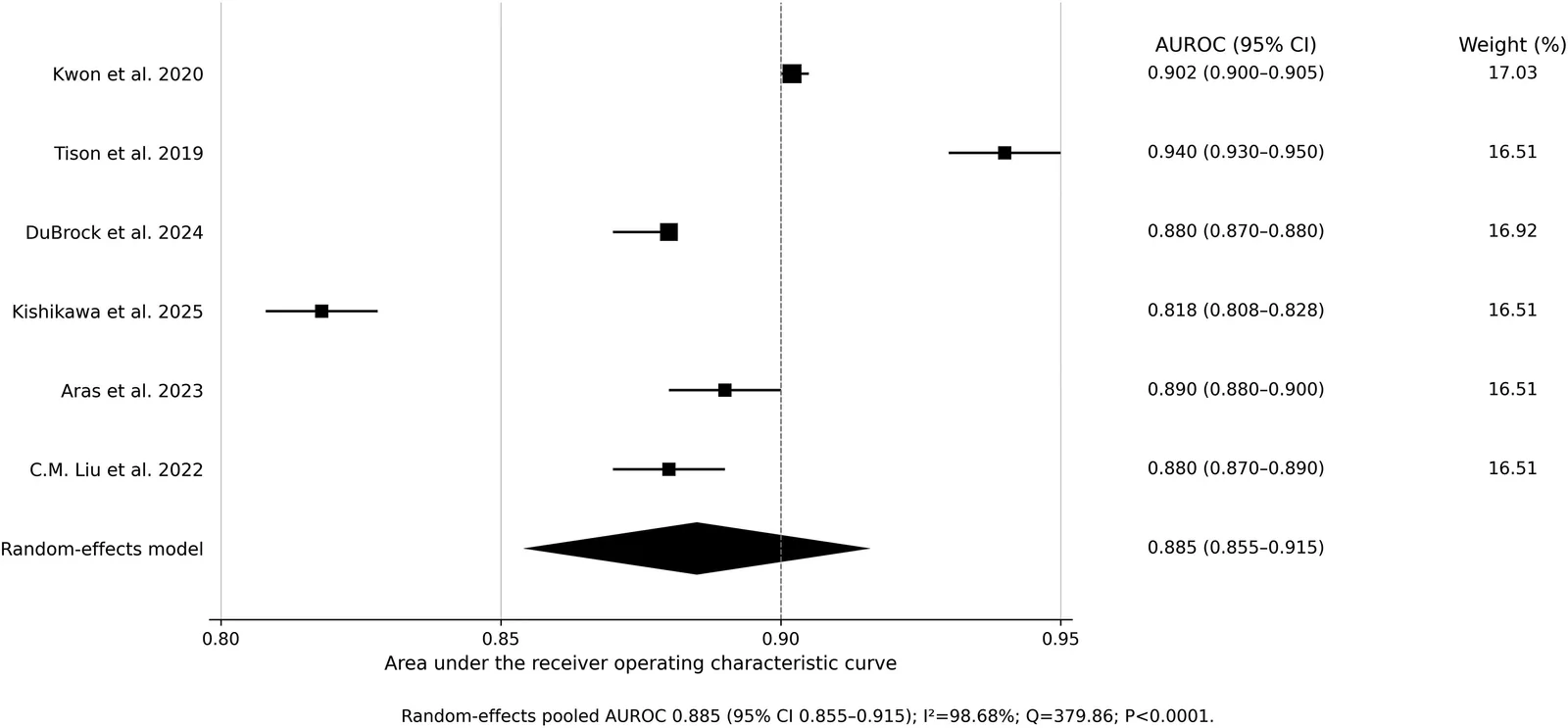
Random-effects meta-analysis of ECG-only AUROC estimates for study-defined pulmonary hypertension-related outcomes. Six studies contributed one ECG-only estimate each. P.Y. Liu *et al*. were excluded from quantitative synthesis because a compatible confidence interval or standard error was not reported. The pooled estimate includes heterogeneous target conditions, including mixed RHC/echocardiographic definitions, echocardiography-defined elevated pulmonary pressure or probability of pulmonary hypertension, and a clinical–echocardiographic pulmonary arterial hypertension phenotype. It should not be interpreted as a diagnostic accuracy for uniformly RHC-confirmed pulmonary hypertension. AUROC, area under the receiver operating characteristic curve; CI, confidence interval; RHC, right-heart catheterization.

### Sensitivity, specificity, and predictive values

Sensitivity and specificity varied according to the model, target definition, cohort, and operating threshold. DuBrock *et al*.^12^ reported sensitivities of 85.5% and 82.3%, with specificities of 83.8% and 77.0%, in the Mayo and external Vanderbilt diagnostic cohorts, respectively. Aras *et al*.^11^ reported sensitivity of ∼78–79% and specificity of 84%. P.Y. Liu *et al*.^13^ reported sensitivity and specificity of 76.6% and 74.5% internally and 78.9% and 73.7% externally. C.M. Liu *et al*.^9^ reported sensitivity of 81.0% and specificity of 79.6%; external validation produced higher sensitivity but lower specificity of 93.9% and 58.7%, respectively. Kwon *et al*.^15^ reported 80% sensitivity in both validation cohorts, with specificity of 74% internally and 84% externally. In Kishikawa *et al*.,^10^ the ECG-only model had a sensitivity of 75.6% and a specificity of 72.5%, whereas the multimodal ensemble had a sensitivity of 74.6% and a specificity of 85.7%.

Where reported, positive predictive values were generally modest, and negative predictive values were high, reflecting the low prevalence of the study-defined target in several cohorts. In Kishikawa *et al*.,^10^ the ECG-only model had a positive predictive value of 28.9% and a negative predictive value of 95.3%; the ensemble increased the positive predictive value to 52.5% while retaining a negative predictive value of 94.1%. Because predictive values are prevalence dependent and were not pooled, these results cannot be transferred directly between populations or used to establish AI–enabled ECG as a stand-alone rule-out test.

### Temporal and subgroup findings

DuBrock *et al*.^12^ evaluated ECGs obtained before recognized PH and reported decreasing discrimination with increasing time before diagnosis: AUROCs at 6–18 months were 0.86 at Mayo and 0.81 at Vanderbilt, with performance remaining at least 0.79 and 0.73, respectively, up to 5 years before diagnosis. Aras *et al*.^11^ reported an AUROC of at least 0.79 using ECGs obtained up to 2 years before the reference assessment. These results demonstrate the detection of a signal preceding recognized diagnosis, but do not establish that the models identify a treatable early disease stage.

Reported subgroup analyses generally showed preserved discrimination across age and sex, although performance varied in some cardiopulmonary comorbidity groups.^11,12^

### Multimodal performance and clinical impact

Two studies assessed multimodal modelling. Kishikawa *et al*.^10^ reported an AUROC of 0.872 for the ECG-chest-radiography-BNP ensemble compared with 0.818 for ECG alone. P.Y. Liu *et al*.^13^ reported an external AUROC of 0.8734 for combined ECG and chest radiography compared with 0.8348 for ECG alone. These findings suggest potential incremental value, but multimodal estimates were excluded from the ECG-only meta-analysis and should be interpreted as separate, hypothesis-generating evidence.

Kishikawa *et al*.^10^ also assessed clinician assistance. Mean cardiologist accuracy increased from 65.0% ± 4.7% without AI support to 74.0% ± 2.7% with AI support (*P* < 0.01), with improvements in sensitivity and specificity.

## Discussion

This systematic review identified seven retrospective studies of AI–enabled ECG for PH-related targets. Six contributed to an ECG-only meta-analysis, producing a pooled AUROC of 0.885. However, heterogeneity was very high, and the target condition was not uniform. Studies evaluated mixed RHC/echocardiographic PH, isolated echocardiographic pressure thresholds, composite echocardiographic probability, and a clinical–echocardiographic PAH phenotype. The pooled estimate should therefore be interpreted as an average of discrimination across related but non-interchangeable outcomes, not as a diagnostic accuracy for uniformly haemodynamically confirmed PH.

AI–enabled models generally showed stronger discrimination than conventional ECG criteria, suggesting that machine-learning methods can identify electrophysiological signatures associated with pulmonary vascular disease and right-heart remodelling that are not apparent to routine interpretation. External validation in four studies provides some evidence of transportability,^9,12,13,15^ but performance was not uniform across settings, and all evidence was retrospective. More broadly, translation of cardiovascular AI models into routine care remains constrained by changes in population case mix, calibration drift, workflow integration, interpretability, and regulatory requirements.^16^

The clinical relevance of echocardiography-labelled studies lies primarily in case finding and triage rather than definitive diagnosis. An AI–ECG output might help identify patients who warrant comprehensive clinical assessment, echocardiography and, when indicated, RHC. However, discrimination of an echocardiographic pressure threshold cannot be equated with the diagnosis of haemodynamically confirmed PH, and AI–enabled ECG cannot replace echocardiographic probability assessment or RHC.

Echocardiographic probability was operationalized differently across studies. Contemporary evaluation does not rely on tricuspid regurgitation velocity or estimated systolic pulmonary artery pressure alone but integrates additional signs of right-heart pressure overload and remodelling, including right-ventricular and right-atrial enlargement, interventricular septal flattening, pulmonary artery dilatation, or flow notching, and inferior vena cava/right-atrial abnormalities.^1^ Most included studies used isolated velocity or pressure thresholds. Kwon *et al*.^15^ instead used a composite definition incorporating estimated pulmonary pressure and, where the tricuspid regurgitation gradient was lower, additional measures of right-ventricular size and function. Consequently, echocardiography-positive groups represented different diagnostic probabilities, severities, and phenotypes, contributing to clinical heterogeneity, spectrum effects, and possible misclassification.

The generally high negative predictive values reported in several cohorts are potentially attractive for triage but should be interpreted cautiously. Predictive values depend on disease prevalence, referral pathways, and the selected operating threshold. The low prevalence of the target condition in several datasets increased negative predictive values while limiting positive predictive values. Prospective implementation studies must therefore evaluate model calibration, threshold selection, net benefit, and downstream testing within the intended population rather than relying on retrospective AUROC or predictive values alone.

Multimodal models yielded higher AUROCs than ECG-only models in two studies.^10,13^ This is biologically and clinically plausible because ECG, chest radiography, and natriuretic peptides capture complementary aspects of the right-heart strain, pulmonary vascular disease, and haemodynamic stress. Nevertheless, these findings arose from retrospective studies and should not be taken as evidence that multimodal AI improves clinical outcomes. The clinician-assistance experiment reported by Kishikawa *et al*.^10^ is encouraging, but prospective trials are required to establish whether AI support shortens diagnostic pathways, reduces missed disease, avoids unnecessary testing, or improves outcomes.

Temporal analyses suggest that AI–enabled ECG may detect a PH-associated signal months or years before a recognized diagnosis.^11,12^ This observation does not establish that the model identifies an actionable preclinical stage. Earlier ECGs may already reflect established comorbidity, right-heart remodelling, or healthcare-utilization patterns. Prospective longitudinal studies linking model output to serial haemodynamic, imaging, and clinical data are needed to determine what these early signals represent and whether acting on them is beneficial.

Methodological reporting also requires improvement. Model architecture, preprocessing, threshold selection, calibration, and patient-level partitioning were not consistently described. Repeated ECGs can increase effective sample size, but failure to keep all examinations from one participant within a single partition may cause information leakage. Most included studies explicitly used patient-level separation or one ECG per patient during validation; however, this was unclear in Tison *et al*.^14^ Future studies should report unique patients separately from ECG examinations, prespecify data partitions, evaluate calibration and clinical utility, and perform independent external validation in populations differing by geography, ethnicity, healthcare setting, and PH aetiology.

## Limitations

This review has several limitations. First, the pooled AUROC combined clinically heterogeneous target conditions. Right-heart catheterization is required for definitive haemodynamic diagnosis, but most studies used echocardiographic surrogate labels and no RHC-only meta-analysis was possible. The summary estimate therefore cannot be interpreted as accuracy for confirmed PH.

Second, echocardiographic definitions were not standardized. Several studies used an isolated tricuspid regurgitation velocity, right ventricular systolic pressure, or estimated pulmonary artery pressure threshold rather than a comprehensive probability assessment incorporating additional right-heart and pulmonary artery signs. This may have produced inconsistent classification and spectrum effects.

Third, all studies were retrospective and susceptible to selection bias, overfitting, and optimistic threshold selection. Although four studies reported independent external validation, prospective real-world validation was absent. Model architectures, input formats, and operating thresholds also differed substantially.

Fourth, sensitivity and specificity could not be synthesized using a bivariate model because compatible 2 × 2 data were unavailable and thresholds varied. Predictive values were prevalence dependent and could not be generalized across settings.

Fifth, several studies included repeated ECGs. Most used patient-level separation or one ECG per patient during validation, but the cross-validation unit was unclear in one study. Residual data leakage or failure to account for within-patient correlation may therefore have inflated precision.

Sixth, the Google Scholar search was restricted to the first 200 relevance-ranked results. Rankings can change over time, and exhaustive reproducible export is unavailable. Despite searching biomedical, multidisciplinary, and engineering databases, some unpublished or poorly indexed reports may have been missed.

Finally, the small evidence base precluded reliable assessment of small-study effects and limited subgroup analysis. Evidence regarding workflow integration, cost-effectiveness, downstream testing, and patient outcomes remains sparse.

## Conclusions

AI–enabled ECG models demonstrated promising discrimination for study-defined PH-related target conditions, with a pooled AUROC of 0.885. However, the summary estimate combined heterogeneous populations, model architectures, and target definitions, including mixed RHC/echocardiographic criteria, echocardiography-defined elevated pulmonary pressure, and clinical–echocardiographic phenotypes. It should not be interpreted as diagnostic accuracy for uniformly haemodynamically confirmed PH.

Current evidence supports further evaluation of AI–enabled ECG as a case finding or triage aid rather than as a definitive diagnostic or stand-alone rule-out test. Future studies should use standardized target definitions, prespecified patient-level partitioning, transparent model reporting, calibration assessment, and independent external validation. Diagnostic-performance studies should preferentially anchor outcomes to RHC or clearly distinguish echocardiographic surrogate labels, while prospective clinical-impact studies are required to determine whether AI–enabled ECG shortens diagnostic pathways or improves patient outcomes.

## Supplementary Material

ztag143_Supplementary_Data

## Data Availability

The extracted study-level data, risk-of-bias assessments, GRADE evidence profiles, analytical classifications, and code used to generate the meta-analyses are provided in the manuscript. Further enquiries may be directed to the corresponding author.

## Supplementary material

The Supplementary material accompanying this manuscript includes Supplementary material online, *File S1*, PRISMA 2020 Checklist, and Supplementary material online, *File S2*, Full electronic search strategies. Supplementary material is available at *European Heart Journal – Digital Health*.
