# Supplementary material for: Diagnostic performance of artificial intelligence–enabled electrocardiography for pulmonary hypertension: a systematic review and meta-analysis
Source: Eur Heart J Digit Health. 2026 Sep 8;7(8):ztag143. doi: 10.1093/ehjdh/ztag143 (PMC13637041; doi:10.1093/ehjdh/ztag143)
Supplement: Supplementary file 1 [file ztag143_supplementary_data.zip › s1 AI PH EHJ.docx]

**Supplementary Table S1. Full electronic search strategies**

| **Database/source** | **Platform** | **Coverage** | **Date searched** | **Limits** | **Full search strategy** | **Records retrieved** |
| --- | --- | --- | --- | --- | --- | --- |
| MEDLINE | PubMed | Database inception to 27 July 2026 | 27 July 2026 | None | ("Hypertension, Pulmonary"[Mesh] OR "pulmonary hypertension"[tiab] OR "pulmonary arterial hypertension"[tiab] OR "elevated pulmonary artery pressure"[tiab] OR "elevated pulmonary arterial pressure"[tiab] OR "pulmonary pressure"[tiab]) AND ("Electrocardiography"[Mesh] OR electrocardiogra*[tiab] OR electrocardiogram*[tiab] OR ECG[tiab] OR EKG[tiab] OR "12-lead ECG"[tiab] OR "12 lead ECG"[tiab]) AND ("Artificial Intelligence"[Mesh] OR "Machine Learning"[Mesh] OR "Deep Learning"[Mesh] OR "Neural Networks, Computer"[Mesh] OR "artificial intelligence"[tiab] OR "machine learning"[tiab] OR "deep learning"[tiab] OR "neural network*"[tiab] OR "convolutional neural network*"[tiab] OR "deep neural network*"[tiab] OR algorithm*[tiab] OR "computer-assisted"[tiab] OR "computer assisted"[tiab]) | 312 |
| Embase | Ovid | Database inception to 27 July 2026 | 27 July 2026 | None | 1. exp pulmonary hypertension/ 2. (pulmonary hypertension or pulmonary arterial hypertension or elevated pulmonary artery pressure or elevated pulmonary arterial pressure or pulmonary pressure).ti,ab,kw. 3. 1 or 2 4. exp electrocardiography/ 5. (electrocardiogra* or electrocardiogram* or ECG or EKG or 12-lead ECG or 12 lead ECG).ti,ab,kw. 6. 4 or 5 7. exp artificial intelligence/ 8. exp machine learning/ 9. exp deep learning/ 10. exp neural network/ 11. (artificial intelligence or machine learning or deep learning or neural network* or convolutional neural network* or deep neural network* or algorithm* or computer-assisted or computer assisted).ti,ab,kw. 12. 7 or 8 or 9 or 10 or 11 13. 3 and 6 and 12 | 418 |
| Web of Science Core Collection | Web of Science | Database inception to 27 July 2026 | 27 July 2026 | None | TS=(("pulmonary hypertension" OR "pulmonary arterial hypertension" OR "elevated pulmonary artery pressure" OR "elevated pulmonary arterial pressure" OR "pulmonary pressure") AND (electrocardiogra* OR electrocardiogram* OR ECG OR EKG OR "12-lead ECG" OR "12 lead ECG") AND ("artificial intelligence" OR "machine learning" OR "deep learning" OR "neural network*" OR "convolutional neural network*" OR "deep neural network*" OR algorithm* OR "computer-assisted" OR "computer assisted")) | 276 |
| Scopus | Scopus | Database inception to 27 July 2026 | 27 July 2026 | None | TITLE-ABS-KEY(("pulmonary hypertension" OR "pulmonary arterial hypertension" OR "elevated pulmonary artery pressure" OR "elevated pulmonary arterial pressure" OR "pulmonary pressure") AND (electrocardiogra* OR electrocardiogram* OR ECG OR EKG OR "12-lead ECG" OR "12 lead ECG") AND ("artificial intelligence" OR "machine learning" OR "deep learning" OR "neural network*" OR "convolutional neural network*" OR "deep neural network*" OR algorithm* OR "computer-assisted" OR "computer assisted")) | 329 |
| IEEE Xplore | IEEE Xplore Digital Library | Database inception to 27 July 2026 | 27 July 2026 | None | (("All Metadata":"pulmonary hypertension" OR "All Metadata":"pulmonary arterial hypertension" OR "All Metadata":"elevated pulmonary artery pressure" OR "All Metadata":"elevated pulmonary arterial pressure") AND ("All Metadata":"electrocardiography" OR "All Metadata":"electrocardiogram" OR "All Metadata":"ECG" OR "All Metadata":"EKG" OR "All Metadata":"12-lead ECG") AND ("All Metadata":"artificial intelligence" OR "All Metadata":"machine learning" OR "All Metadata":"deep learning" OR "All Metadata":"neural network" OR "All Metadata":"convolutional neural network" OR "All Metadata":"deep neural network" OR "All Metadata":"algorithm")) | 105 |
| Google Scholar | Google Scholar | Supplementary cross-disciplinary search | 27 July 2026 | First 200 results sorted by relevance; no date or language restriction | ("pulmonary hypertension" OR "pulmonary arterial hypertension") (electrocardiogram OR electrocardiography OR ECG OR EKG) ("artificial intelligence" OR "machine learning" OR "deep learning" OR "neural network" OR "convolutional neural network") | 200 |
| **Total** | **—** | **—** | **—** | **—** | **—** | 1,640 |

The combined searches identified 1,640 records before duplicate removal. Google Scholar was used as a supplementary cross-disciplinary source, with the first 200 results ranked by relevance screened.

Abbreviations: ECG, electrocardiogram/electrocardiography; EKG, electrocardiogram; IEEE, Institute of Electrical and Electronics Engineers; MeSH, Medical Subject Headings; PH, pulmonary hypertension.
