# Supplementary material for: Diagnostic performance of artificial intelligence–enabled electrocardiography for pulmonary hypertension: a systematic review and meta-analysis
Source: Eur Heart J Digit Health. 2026 Sep 8;7(8):ztag143. doi: 10.1093/ehjdh/ztag143 (PMC13637041; doi:10.1093/ehjdh/ztag143)
Supplement: Supplementary file 1 [file ztag143_supplementary_data.zip › Supplementary File 2 Search Stratergy.docx]

**Supplementary File 2- Full electronic search strategies**

**MEDLINE via PubMed**

| **Platform** | PubMed |
| --- | --- |
| **Coverage** | Database inception to 27 July 2026 |
| **Date searched** | 27 July 2026 |
| **Limits** | None |

```
(
  "Hypertension, Pulmonary"[Mesh]
  OR "pulmonary hypertension"[tiab]
  OR "pulmonary arterial hypertension"[tiab]
  OR "elevated pulmonary artery pressure"[tiab]
  OR "elevated pulmonary arterial pressure"[tiab]
  OR "pulmonary pressure"[tiab]
)
AND
(
  "Electrocardiography"[Mesh]
  OR electrocardiogra*[tiab]
  OR electrocardiogram*[tiab]
  OR ECG[tiab]
  OR EKG[tiab]
  OR "12-lead ECG"[tiab]
  OR "12 lead ECG"[tiab]
)
AND
(
  "Artificial Intelligence"[Mesh]
  OR "Machine Learning"[Mesh]
  OR "Deep Learning"[Mesh]
  OR "Neural Networks, Computer"[Mesh]
  OR "artificial intelligence"[tiab]
  OR "machine learning"[tiab]
  OR "deep learning"[tiab]
  OR "neural network*"[tiab]
  OR "convolutional neural network*"[tiab]
  OR "deep neural network*"[tiab]
  OR algorithm*[tiab]
  OR "computer-assisted"[tiab]
  OR "computer assisted"[tiab]
)
```

**Embase via Ovid**

| **Platform** | Ovid |
| --- | --- |
| **Coverage** | Database inception to 27 July 2026 |
| **Date searched** | 27 July 2026 |
| **Limits** | None |

1. exp pulmonary hypertension/
2. (pulmonary hypertension or pulmonary arterial hypertension or elevated pulmonary artery pressure or elevated pulmonary arterial pressure or pulmonary pressure).ti,ab,kw.
3. 1 or 2
4. exp electrocardiography/
5. (electrocardiogra* or electrocardiogram* or ECG or EKG or 12-lead ECG or 12 lead ECG).ti,ab,kw.
6. 4 or 5
7. exp artificial intelligence/
8. exp machine learning/
9. exp deep learning/
10. exp neural network/
11. (artificial intelligence or machine learning or deep learning or neural network* or convolutional neural network* or deep neural network* or algorithm* or computer-assisted or computer assisted).ti,ab,kw.
12. 7 or 8 or 9 or 10 or 11
13. 3 and 6 and 12

**Web of Science Core Collection**

| **Platform** | Web of Science |
| --- | --- |
| **Database** | Web of Science Core Collection |
| **Coverage** | Database inception to 27 July 2026 |
| **Date searched** | 27 July 2026 |
| **Limits** | None |

```
TS=(
  ("pulmonary hypertension" OR "pulmonary arterial hypertension" OR
   "elevated pulmonary artery pressure" OR "elevated pulmonary arterial pressure" OR
   "pulmonary pressure")
  AND
  (electrocardiogra* OR electrocardiogram* OR ECG OR EKG OR
   "12-lead ECG" OR "12 lead ECG")
  AND
  ("artificial intelligence" OR "machine learning" OR "deep learning" OR
   "neural network*" OR "convolutional neural network*" OR
   "deep neural network*" OR algorithm* OR "computer-assisted" OR
   "computer assisted")
)
```

**Scopus**

| **Platform** | Scopus |
| --- | --- |
| **Coverage** | Database inception to 27 July 2026 |
| **Date searched** | 27 July 2026 |
| **Limits** | None |

```
TITLE-ABS-KEY(
  ("pulmonary hypertension" OR "pulmonary arterial hypertension" OR
   "elevated pulmonary artery pressure" OR "elevated pulmonary arterial pressure" OR
   "pulmonary pressure")
  AND
  (electrocardiogra* OR electrocardiogram* OR ECG OR EKG OR
   "12-lead ECG" OR "12 lead ECG")
  AND
  ("artificial intelligence" OR "machine learning" OR "deep learning" OR
   "neural network*" OR "convolutional neural network*" OR
   "deep neural network*" OR algorithm* OR "computer-assisted" OR
   "computer assisted")
)
```

**IEEE Xplore**

| **Platform** | IEEE Xplore Digital Library |
| --- | --- |
| **Coverage** | Database inception to 27 July 2026 |
| **Date searched** | 27 July 2026 |
| **Limits** | None |

```
(
  ("All Metadata":"pulmonary hypertension" OR
   "All Metadata":"pulmonary arterial hypertension" OR
   "All Metadata":"elevated pulmonary artery pressure" OR
   "All Metadata":"elevated pulmonary arterial pressure")
  AND
  ("All Metadata":"electrocardiography" OR
   "All Metadata":"electrocardiogram" OR
   "All Metadata":"ECG" OR
   "All Metadata":"EKG" OR
   "All Metadata":"12-lead ECG")
  AND
  ("All Metadata":"artificial intelligence" OR
   "All Metadata":"machine learning" OR
   "All Metadata":"deep learning" OR
   "All Metadata":"neural network" OR
   "All Metadata":"convolutional neural network" OR
   "All Metadata":"deep neural network" OR
   "All Metadata":"algorithm")
)
```

**Google Scholar**

| **Platform** | Google Scholar |
| --- | --- |
| **Role** | Supplementary cross-disciplinary search |
| **Date searched** | 27 July 2026 |
| **Screening** | First 200 results sorted by relevance |
| **Limits** | No date or language restriction |

```
("pulmonary hypertension" OR "pulmonary arterial hypertension")
(electrocardiogram OR electrocardiography OR ECG OR EKG)
("artificial intelligence" OR "machine learning" OR "deep learning" OR
 "neural network" OR "convolutional neural network")
```
